# Targeting Amyloids with [^18^F]AV-45 for Medullary Thyroid Carcinoma Positron Emission Tomography/Computed Tomography Imaging: A Pilot Clinical Study

**DOI:** 10.1021/acs.molpharmaceut.1c00680

**Published:** 2022-01-04

**Authors:** Chun Li, Pengxin Zhang, Ruirui Nie, Xiaoyan Gong, Jinghui Xie, Zilin Yu, Chengdong Wang, Hua Zhang, Ran Yan, Zhi Lu

**Affiliations:** † Department of Nuclear Medicine, First Affiliated Hospital of Dalian Medical University, Liaoning 116021, People’s Republic of China; ‡ Department of Pathology, First Affiliated Hospital of Dalian Medical University, Liaoning 116021, People’s Republic of China; § School of Biomedical Engineering and Imaging Sciences, King’s College London, St. Thomas’ Hospital, London SE1 7EH, U.K.

**Keywords:** medullary thyroid carcinoma, [^18^F]AV-45, amyloid, PET/CT

## Abstract

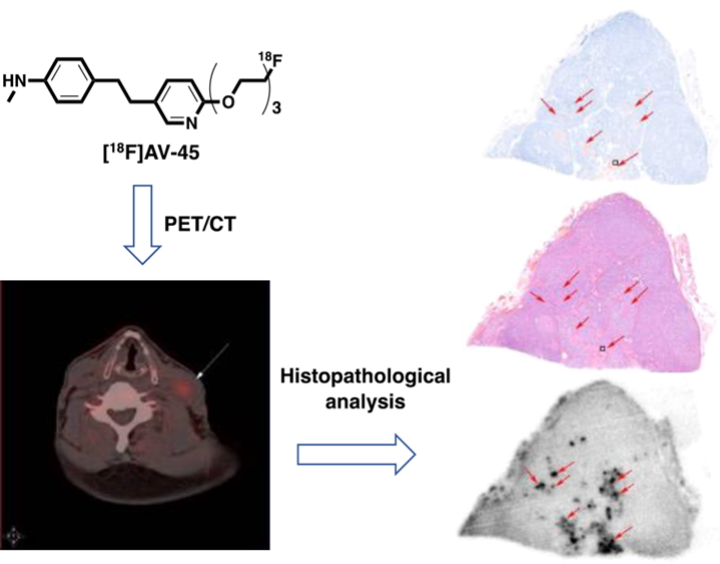

Medullary thyroid carcinoma (MTC) is a malignant neuroendocrine tumor with a high recurrence rate. Amyloid plaques formed from the misfolding of calcitonin are the key characteristics of MTC. Herein, we conducted a first-in-human pilot clinical study by applying a β-amyloid-specific radiotracer, [^18^F]AV-45, to positron emission tomography (PET)/computed tomography (CT) imaging of MTC. The presence of amyloid plaques in the tumor tissue sections from five MTC patients was confirmed by hematoxylin and eosin (H&E) and Congo Red staining. [^18^F]AV-45 selectively accumulated in the amyloid plaques in the continued tumor tissue sections with similar distribution patterns to the H&E and Congo Red staining. In addition, the [^18^F]AV-45 uptake can be largely blocked by its nonradioactive reference compound. The [^18^F]AV-45 accumulation in the thyroid, neck lymph nodes, and muscles in healthy human subjects is close to the background indicated by PET/CT imaging. In the comparison PET/CT imaging study of a recurrent MTC patient, 2-deoxy-2-[^18^F]fluoro-d-glucose ([^18^F]FDG) showed an elevated uptake by multiple neck lymph nodes. In contrast, only one of these neck lymph nodes had increased [^18^F]AV-45 uptake. Postoperative histopathological analysis confirmed the [^18^F]AV-45 PET-positive lymph node as MTC with amyloid deposition, while other [^18^F]FDG positive lymph nodes were free from MTC and amyloid plaques. Thus, [^18^F]AV-45 showed the promise for the clinical PET/CT imaging of MTC.

## Introduction

Medullary thyroid carcinoma (MTC) is a type of malignant neuroendocrine tumor derived from parafollicular C cells in the thyroid gland.^1^ Although it only accounts for around 1∼2% of thyroid cancers, MTC causes 13.4% of all thyroid-related deaths.^2^ Nearly 50% of MTC patients develop recurrent lesions, and the prognosis is closely related to the MTC development stage.^3^ American Joint Committee on Cancer (AJCC) stage I, II, and III MTC patients have 10-year survival rates of 100, 93, and 71%, respectively, but the rate drastically decreased to 21% in AJCC stage IV MTC patients. Therefore, early diagnosis is vital for the survival of MTC patients.^3^

Currently, the invasive fine-needle aspiration cytology (FNAC) is still the gold standard of MTC diagnosis, with a detection rate of about 50%.^4^ Conventional anatomic imaging techniques, such as neck ultrasonography (US), contrast-enhanced computed tomography (CT), and magnetic resonance imaging (MRI), are used to detect MTC metastatic lesions.^5^ However, it is challenging for these morphological imaging modalities to visualize lymph node lesions and small liver metastasis.^5^ Several single-photon emission computed tomography (SPECT) tracers, such as [^99m^Tc](V)-dimercaptosuccinic acid (DMSA), [^123^I]-I-meta-iodobenzylguanidine (MIBG), and [^111^In]-pentetreotide, have been used to image recurrent MTC but failed to provide sufficient sensitivity to localize metastatic lesions.^6,7^

Positron emission tomography (PET) used for measuring cancer-associated biochemical variations is considered a superior functional imaging technique, offering a higher spatial resolution and better image quality of malignant lesions. Several MTC PET imaging comparison studies with 2-deoxy-2-[^18^F]fluoro-d-glucose ([^18^F]FDG), 3,4-dihydroxy-6-[^18^F]fluoro-l-phenylalanine ([^18^F]FDOPA), and [^68^Ga]-somatostatin conclude that [^18^F]FDOPA measuring the increased activity of L-type amino-acid transporter 1 has the best diagnostic performance among these three PET tracers.^8,9^ [^18^F]FDOPA has been recommended by the European Association of Nuclear Medicine (EANM) as the first-line procedure for the diagnosis of MTC with PET, if available.^10^ However, the availability of [^18^F]FDOPA is limited to only a handful of PET centers worldwide because of its challenging radiosynthesis, prolonged preparation time, and low radiochemical yields.^11^

Amyloid deposition is associated with many pathologically unrelated human diseases such as Alzheimer’s, Parkinson’s, and Huntington’s diseases, as well as MTC.^12,15^ Although originated from the misfolding of different proteins, these unrelated amyloid plaques share a similar β-sheet structure.^13,15^ MTC contains amyloid deposition formed from misfolded calcitonins that are rich in β-sheet structures.^15^ Thus, we hypothesize that PET tracers such as [^18^F]AV-45 (florbetapir)^14^ for imaging amyloid burden in Alzheimer’s disease can be applied to MTC diagnosis. In this first-in-human clinical study, we report (i) fully automated preparation and quality control of clinical-grade [^18^F]AV-45; (ii) [^18^F]AV-45 had specific uptake by the amyloid plaque in postoperative MTC tissue; (iii) [^18^F]AV-45 PET/CT successfully detected metastatic lymph lesions in a recurrent MTC patient. [^18^F]FDG PET/CT gave false-positive diagnosis of several lymph lesions in the same patient.

## Experimental Section

### General Information

Paraffin-embedded human MTC tissue samples from six patients and paraffin-embedded healthy human thyroid tissue samples were generously provided by the Department of Pathology, the First Affiliated Hospital of Dalian Medical University. The MTC samples were cut into 3∼5 μm-thick sections and used immediately after deparaffinization. The presence of amyloid plaque was determined by two senior pathologists independently. Fluoride-18 was produced by a Seimens Eclipse RD Cyclotron. [^18^F]AV-45 was prepared using a Trasis Allinone automated synthesizer. PET/CT imaging was acquired using a Seimens Biograph64 PET/CT scanner.

### Ethics Approval and Consent To Participate

Ethics approval (YJ-KY-FB-2020-26) for using patients’ imaging data and postsurgical tissue was obtained from the Institute Research Medical Ethics Committee of the First Affiliated Hospital of Dalian Medical University. The patient involved in this study had given a written consent.

### Human Participants

Four volunteers aged between 42 and 65 without thyroid disease history participated in the [^18^F]AV-45 PET/CT imaging study as the control group. One recurrent MTC patient (62-year-old male) who had previously received thyroidectomy participated in the [^18^F]FDG and [^18^F]AV-45 PET/CT imaging comparison study.

### Automated Preparation, Purification, Formulation, and Quality Control of [^18^F]AV-45

In a Trasis Allinone automated synthesizer, fluoride-18 (∼40 GBq) was trapped on a QMA cartridge (preactivated with 10 mL of 1.0 M NaHCO_3_ and 10 mL of sterilized water) and then eluted with a solution of Kryptofix222 (25 mg)/K_2_CO_3_ (5 mg) in AcCN/H_2_O (4/1 v/v, 0.5 mL) to the reaction vial. Water was removed by two rounds of azeotropic distillation with anhydrous AcCN (2 × 0.5 mL) at 110 °C. A solution of the precursor, AV-105 (1 mg) in anhydrous dimethyl sulfoxide (DMSO) (1.0 mL) was added to the reaction vial and heated at 110 °C for 10 min. The reaction was cooled to 60 °C before the addition of 3 M HCl (0.8 mL) for deprotection. The reaction was heated at 120 °C for 5 min and cooled to room temperature (RT), to which 1 M NaOH (2.5 mL) and sodium ascorbate (6.5 mL, 5 mg/mL) were added to neutralize to pH 7.0. The crude reaction mixture was purified by a built-in liquid chromatography using a C18-HPLC column (Waters XBridge Prep, 5 μm, 4.6 × 150 mm). The mobile phase is AcCN/H_2_O (10: 9) containing sodium ascorbate (5 mg/mL) and NH_4_OAc (0.73 mg/mL) with a flow rate of 5 mL/min. The retention time of [^18^F]AV-45 is 6.5 min. The [^18^F]AV-45 was diluted with saline (20 mL), trapped on a tC18 cartridge, and released with ethanol (1 mL) into a collection vial containing saline (10 mL) with sodium ascorbate (5 mg/mL). The solution was filtered using a 0.22 μm Millipore sterile filter into a sterile vial for injection. The chemical and radiochemical purity and the molar activity of [^18^F]AV-45 were determined using an Agilent 1200 HPLC with a Raytest GABI Star radioactivity detector, a diode array ultraviolet (UV) detector, and a ZORBAX Eclipse HPLC column (XDB-C18, 4.6 × 150 mm, 5 μm) with a mobile phase of AcCN/H_2_O (11: 9) and a flow rate of 1.0 mL/min. Kryptofix was determined using the kryptofix spot test with silica-based thin-layer chromatography (TLC). The discoloration during TLC was compared with a kryptofix reference solution (25 mg/mL). Radionuclide purity was determined using a germanium detector. Radionuclide identity is determined for the gamma spectrum emitted by the [^18^F]AV-45. The half-life of the radionuclide was measured by the radioactive decay of [^18^F]AV-45 over time. The solvent residual was determined by gas chromatography. Sterility tests were performed by adding the decayed [^18^F]AV-45 to tryptic soy broth (TBS) medium (Soyabean casein digest) for 2 weeks at 25 °C. Bacterial endotoxin was determined using endotoxin assay kits (Genescript).

### Hematoxylin and Eosin Staining

The dewaxed and hydrated human MTC tissue sections were incubated with alum hematoxylin solution at RT for 5 min before sequentially rinsing with distilled water for 1 min, 1% HCl in ethanol for 20 s, and distilled water for 1 min. The sections were then incubated with 0.5% eosin at RT for 2 min, rinsed with distilled water for 1 min, dehydrated, and mounted with glycerol before observation under a Nikon Eclipse E600 microscope with a Nikon DXM 1200 digital camera (Nikon, JP).

### Congo Red Staining

The dewaxed and hydrated human MTC tissue sections were incubated with alum hematoxylin solution at RT for 2 min and then in 0.5% HCl in ethanol for 20 s before rinsing with distilled water twice for 5 min. The sections were then incubated in 1% Congo Red solution at RT for 25 min, rinsed with distilled water for 2 min, dehydrated, and mounted with glycerol before observation under a Nikon Eclipse E600 microscope with a Nikon DXM 1200 digital camera (Nikon, JP).

### [^18^F]AV-45 Autoradiography and Blocking Study

The dewaxed and hydrated human MTC tissue sections were incubated in [^18^F]AV-45 PBS solution (0.37 MBq/mL) with or without its nonradioactive reference compound (125 μg/mL) for 40 min at room temperature. The sections were then washed with distilled water and air-dried prior to exposure to a multisensitive phosphor screen (PerkinElmer AQ5) at RT for 1 h. The phosphor screen was then scanned in a GE Amersham Typhoon Biomolecular Imager at a resolution of 25 μm. The images were analyzed with ImageQuant TL 8.1 software.

### [^18^F]AV-45 PET/CT Imaging of Healthy Human Subjects

Four healthy volunteers (42–65 year old) free from thyroid diseases were intravenously injected [^18^F]AV-45 (∼300 MBq) and rested for 1 h before PET/CT scan. For the anatomic correlation and attenuation correction of PET images, a low-dose CT (120 kV, 35∼170 mAs) was acquired from the vertex of the skull to the proximal femora. Subsequently, a PET scan of the same area was acquired with 2 min per bed position over seven bed positions.

### [^18^F]FDG and [^18^F]AV-45 PET/CT Imaging of an MTC Patient

An MTC patient (62 year old, male) was intravenously injected with [^18^F]FDG in a dose of 5.55 MBq/kg after 6 h of fasting and then rested for 1 h before PET/CT scan. The patient’s blood sugar level was measured as 4.9 mM. Four days later, the same patient received [^18^F]AV-45 (278 MBq) and rested for 1 h before the PET/CT scan. For anatomic correlation and attenuation correction of PET images, a low-dose CT (120 kV, 35∼170 mAs) was acquired from the vertex of the skull to the proximal femora. Subsequently, a PET scan of the same area was acquired with 2 min per bed position over seven bed positions. The images were reconstructed using True X with a 168 × 168 matrix size, ordered-subset expectation maximization (three iterations, 21 subsets, zoom 1) and postfilter full width at half maximum of 4 mm. Quantitative image analysis was based on the transaxial frames of [^18^F]FDG or [^18^F]AV-45 series images by drawing the region of interest (ROI) and then measuring the maximum standardized uptake values (SUVmax) of radioactivity uptake in the neck. On the DICOM reconstructed images, an ROI in the neck was used to measure the lymph nodes and muscle SUVs.

## Results

### [^18^F]AV-45 Automated Radiosynthesis, Purification, Formulation, and Quality Control

**Scheme 1:**
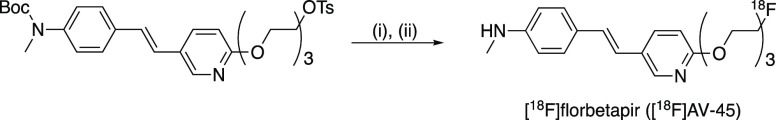
Radiosynthesis of [^18^F]AV-45 (i) ^18^F, K_222_, DMSO, 110 °C, 10 min; (ii) 3 M HCl, 120 °C, 5 min

The fully automated radiosynthesis of [^18^F]florbetapir ([^18^F]AV-45) was achieved from the ^18^F-labeling of AV-105 using a one-pot two-step procedure on a Trasis Allinone synthesizer (Scheme 1, Figures S1 and S2 for a photograph of Trasis Allinone automated synthesizer and a photograph of [^18^F]-AV45 radiosynthesis module control diagram). The overall preparation time was within 60 min from the end of bombardment till formulation. The nondecay correct radiochemical yields of [^18^F]AV-45 were 23.7 ± 1.2% (*n* = 3) with radiochemical purity >99% (Supporting Information Figure S3 for QC HPLC chromatogram). The molar activity of [^18^F]AV-45 was 401 ± 84 GBq/μmoL (*n* = 3) when starting with around 40 GBq of fluoride-18. The purified [^18^F]AV-45 was formulated in injection saline containing 5 mg/mL of sodium ascorbate and 10% ethanol with pH around 6.5. The gamma energy and radionuclide purity of [^18^F]AV-45 were 511 keV and 99%, respectively. The half-life was measured as 108 min. Kryptofix222 was <25 mg/L in the final formulation. The [^18^F]AV-45 formulation was sterile, and endotoxin was <−0.006 EU/mL.

### [^18^F]AV-45 Detected the Amyloid Plaques in MTC Tissue Sections

**Figure 1:**
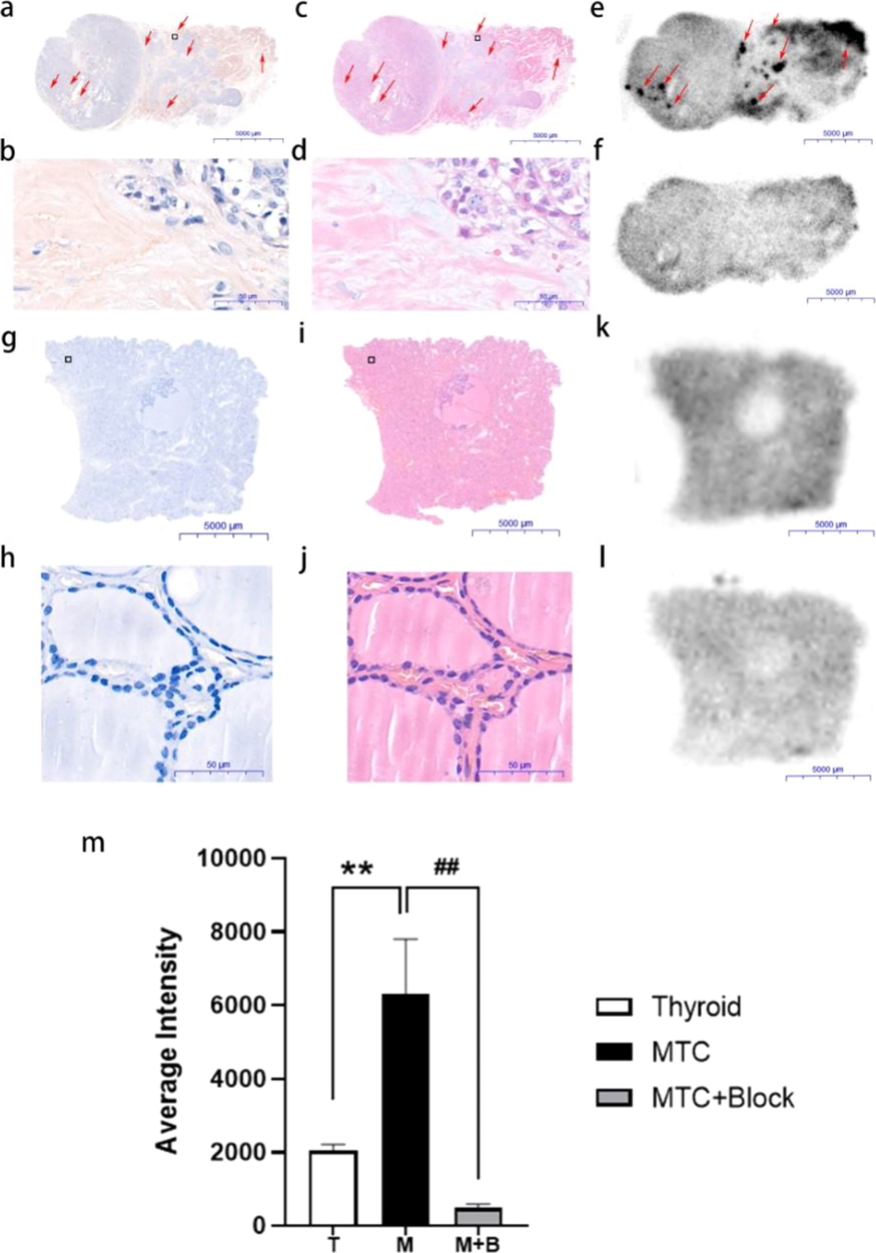
Representative images of continued tissue sections from an MTC tumor (a–f) and a healthy thyroid (g–l). (a,g) Congo Red staining; (b,h) 100× magnification of Congo Red staining; (c,i) H&E staining; (d,j) 100× magnification of H&E staining; (e,k) [^18^F]AV-45 in vitro autoradiography; (f,l) [^18^F]AV-45 in vitro autoradiography with coincubation with the nonradioactive reference compound; (m) the average radioactivity intensity in tissue sections of healthy thyroids, and MTC and MTC with blocking from five patients, **p < 0.01 and ^##^*p* < 0.01. Images and data are representative of five independent experiments.

To confirm the presence of amyloids in MTC, continued tumor sections from five MTC patients excised by surgery were stained with Congo Red and hematoxylin and eosin (H&E). Healthy human thyroid tissue sections were also stained accordingly as the negative control. Amyloid plaques were detected in all five tumor specimens as brown clusters by Congo Red and as pink clusters by H&E under a bright-field microscope indicated by red arrows (Figure 1a,b and c,d). No amyloid deposition was observed in the healthy human thyroid tissue sections (Figure 1g,h and i,j). Next, we investigated the selectivity and specificity of [^18^F]AV-45 to bind to the MTC amyloid plaques in the continued tumor sections from the same five MTC patients by autoradiography. Continued healthy human thyroid tissue sections from the same specimens were also incubated with [^18^F]AV-45 as the negative control. The corresponding blocking experiments were conducted in the presence of the nonradioactive reference compound of [^18^F]AV-45. Localized radioactivity accumulation was observed in the continued MTC tumor sections in the autoradiography images. The distribution of radioactivity is in good agreement with the amyloid plaques detected by the Congo Red and H&E staining (Figure 1e). The selective radioactivity accumulation in the amyloid plaques was largely blocked by the nonradioactive reference compound of [^18^F]AV-45 (Figure 1f). In contrast, there was little specific uptake of [^18^F]AV-45 by the healthy human thyroid tissue sections (Figure 1k,l). Subsequently, the [^18^F]AV-45 uptake by the healthy human thyroid tissue sections and MTC tissue sections without or with blocking was quantified. The average [^18^F]AV-45 uptake by the MTC tissue sections is 3.09- and 13.17-fold of those by the healthy human thyroid tissue sections and by the MTC tissue sections in the blocking experiments, respectively (Figure 1m).

### [^18^F]AV-45 PET/CT Imaging of Healthy Human Subjects

**Figure 2:**
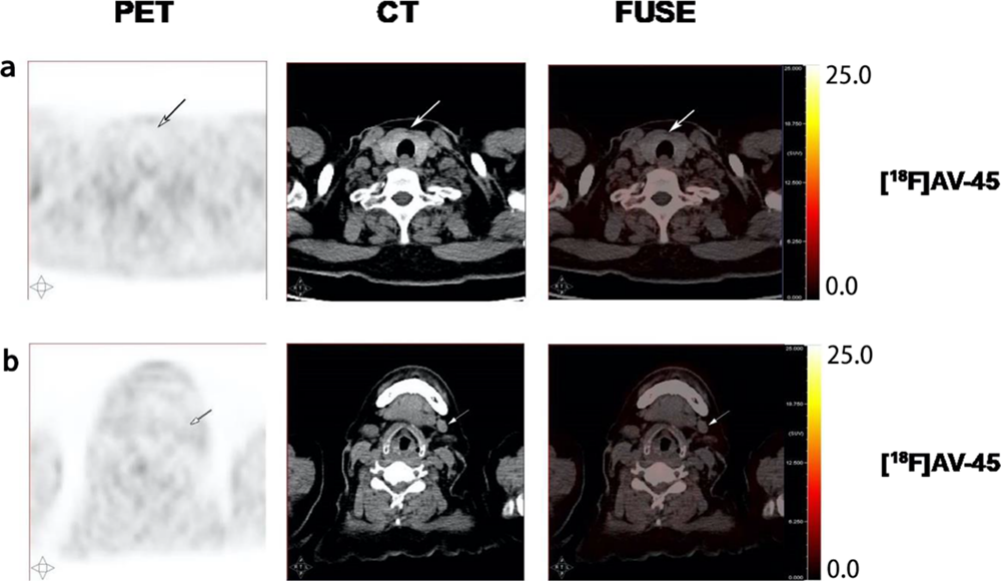
[^18^F]AV-45 PET/CT images of a healthy human subject. Images are representative of four healthy volunteers. (a) Thyroid; (b) lymph nodes.

Four human volunteers free from thyroid diseases were subjected to the [^18^F]-AV-45 PET/CT scan. The SUVmax values of thyroids, neck lymph nodes, and neck muscle were determined and are summarized in Table 1. The thyroid SUVmax values are close to 1.0. The neck lymph nodes SUVmax values are between 0.70 and 1.50. The neck muscle SUVmax values range from 0.90 to 1.33. No specific [^18^F]-AV-45 retention was observed in these tissues (Figure 2).

**Table 1:** Characteristics of Healthy Human Subjects in the [^18^F]-AV-45 PET Imaging Study

	age (years)	gender	thyroid SUVmax	neck lymph node 1 SUVmax	neck lymph node 2 SUVmax	neck muscle SUVmax
1	43	M	1.07	0.97	0.98	1.07
2	65	F	0.97	1.50	1.30	1.33
3	56	M	0.90	1.50	1.20	0.90
4	42	M	1.10	0.70	0.80	1.20

### Comparison of ^18^F-FDG and [^18^F]AV-45 PET/CT Imaging of an MTC Patient with Recurrent Neck Lymph Node Metastasis

**Figure 3:**
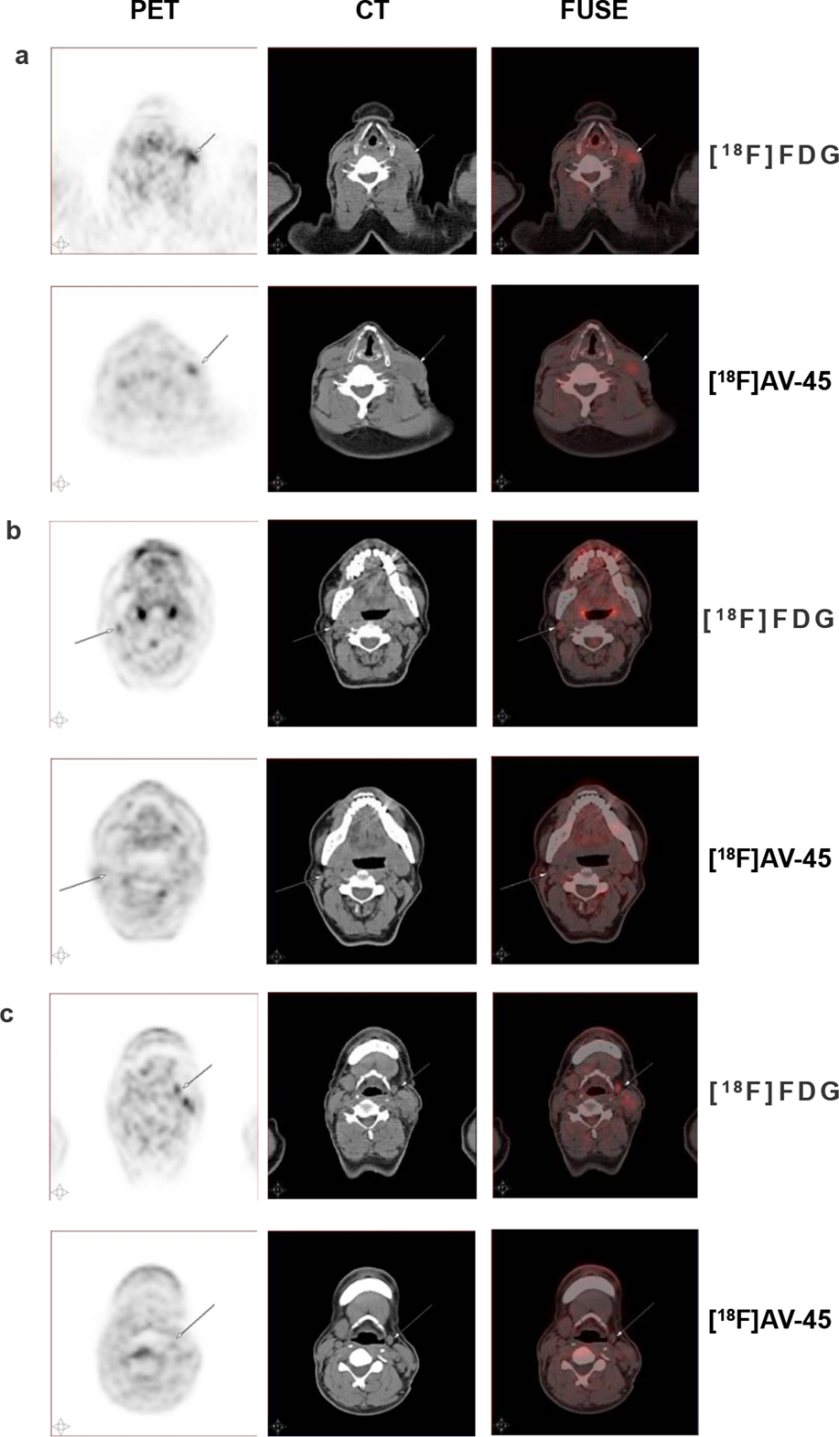
[^18^F]FDG and [^18^F]AV-45 PET/CT images of the MTC patient with recurrent neck lymph node metastasis. (a) MTC-positive lymph node; (b,c) MTC-negative lymph nodes.

The calcitonin level (normal value < 18.2 pg/mL) of the 62-year-old male patient with MTC history was increased to 4246 pg/mL. The [^18^F]FDG PET/CT imaging detected elevated radioactivity uptake by multiple neck lymph nodes with SUVmax values of 4.9, 3.4, and 2.8, indicated by the arrows (Figure 3a–c). In contrast, only one of these lymph nodes showed increased [^18^F]AV-45 uptake with an SUVmax value of 2.5 and the other two [^18^F]FDG positive lymph nodes had [^18^F]AV-45 uptake close to the background level with SUVmax values of 0.9 and 1.0, respectively, in the PET/CT scan indicated by the arrows (Figure 3a–c). However, the radioactivity distribution patterns in the ^18^F-FDG and [^18^F]AV-45 positive lymph nodes were different (Figure 3a). The patient subsequently received a radical neck dissection to remove all the left side neck lymph nodes that were then analyzed by histopathology. The patient’s calcitonin level was reduced to 326 pg/mL 2 months postsurgery and with no detectable disease at the time of this report.

### Postoperative Histopathological Analysis Confirmed [^18^F]AV-45 PET-Positive Lymph Node as MTC with Amyloid Plaques

**Figure 4:**
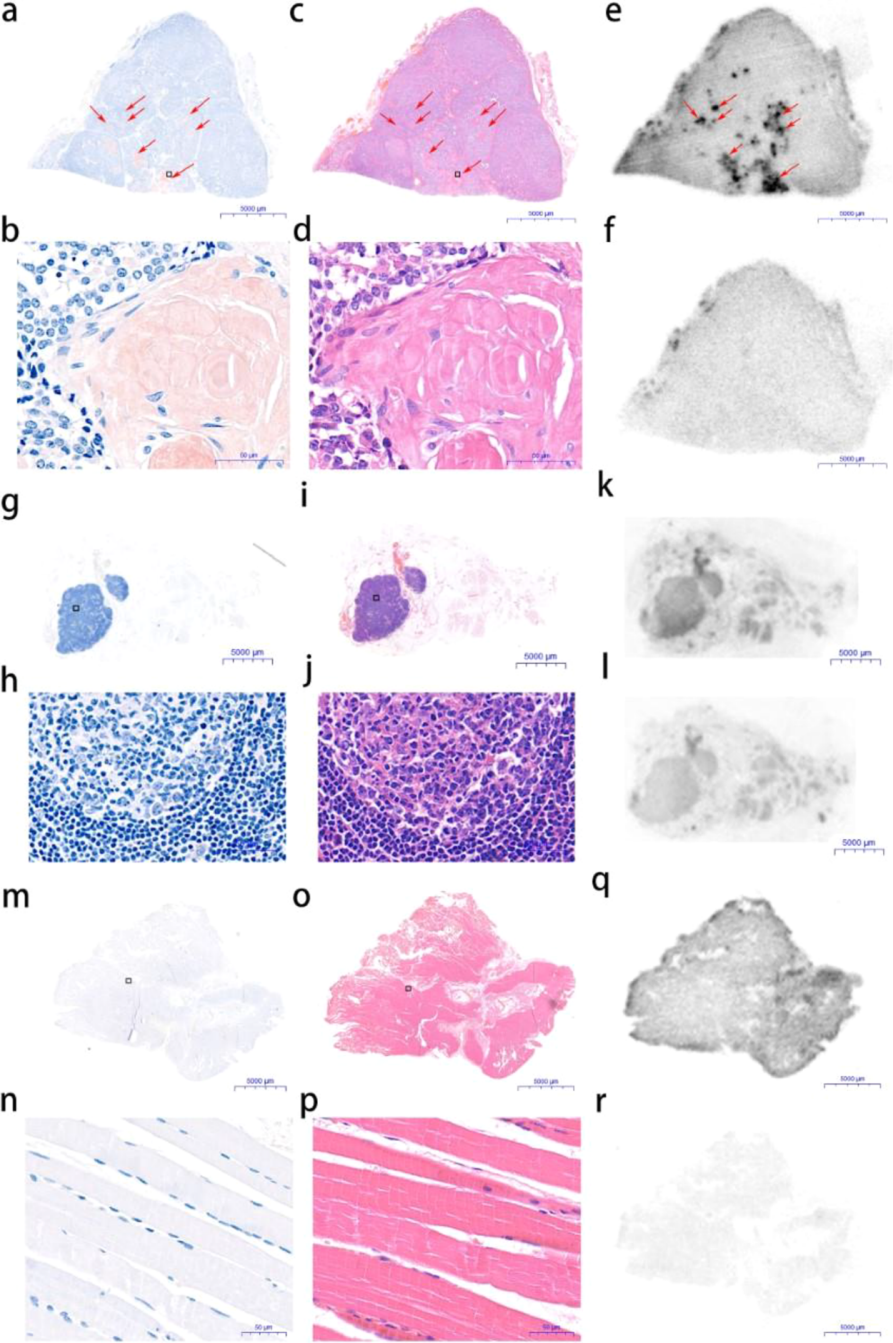
Postoperative histopathological analysis of the metastatic lymph node (a–f), nonmetastatic lymph nodes (g–l), and paratumor muscle tissue sections (m–r) from the MTC patient of [^18^F]AV-45 PET imaging. (a,g,m) Congo Red staining; (b,h,n) 100× magnification of Congo Red staining; (c,i,o) H&E staining; (d,j,p) 100× magnification of H&E staining; (e,k,q) [^18^F]AV-45 autoradiography; and (f,l,r) [^18^F]AV-45 autoradiography with coincubation of the nonradioactive reference compound. Images are representative of three independent experiments.

The excised tissues from the MTC patient, including all left-side cervical lymph nodes and adhesion muscle, were subjected to histopathological examination by Congo Red and H&E staining. The [^18^F]AV-45 PET-positive lymph node was confirmed as MTC, in which amyloid plaques were observed (Figure 4a–d). The [^18^F]AV-45 PET-negative lymph nodes and adhesion muscle were MTC-negative and free from amyloid plaques (Figure 4g–j and m–p). Ex vivo [^18^F]AV-45 autoradiography and the corresponding blocking study were also performed with the continued tissue sections of the above excised tissues. Localized radioactivity uptake was only observed in the [^18^F]AV-45 PET-positive lymph node tissue sections (Figure 4e). The distribution of the radioactivity uptake was correlated with the amyloid plaques detected by both Congo Red and H&E staining. Additionally, the radioactivity uptake was largely blocked by the nonradioactive reference compound of [^18^F]AV-45 (Figure 4f). While the [^18^F]AV-45 uptake by the lymph nodes and paratumor muscle sections free from MTC was minimal (Figure 4k,q).

## Discussion

Despite its rarity, MTC is the most fatal thyroid cancer with a high recurring rate.^2^ The early diagnosis of MTC can significantly improve patients’ prognosis.^3^ Recently, [^18^F]FDOPA has shown superior sensitivity and selectivity toward recurrent MTC.^10^ However, the challenging radiochemical preparation of [^18^F]FDOPA severely limited its clinical availability.^11^ Thus, an alternative PET tracer that can be readily prepared on a standard automated synthesizer is required. Amyloid deposition has been recognized as a characteristic pathology of MTC. The main composition of MTC amyloid is formed from the misfolding of calcitonin that contains β-sheets in its secondary structure,^15^ which makes it an attractive target for molecular imaging. In recent studies, β-amyloid targeting radiotracers such as [^18^F]AV-45 and [^18^F]FDDNP for AD PET imaging have been demonstrated as promising contrast reagents for the detection of cardiac amyloidosis^16^ and pancreatic islet amyloid,^17^ respectively. [^18^F]AV-45 is a specific molecular probe for β-amyloid, while [^18^F]FDDNP binds to both β-amyloid and tangled tau proteins.^18^ In addition, in our laboratory, we have implemented the fully automated preparation of clinical-grade [^18^F]AV-45 with good and reproducible radiochemical yields (RCYs). Thus, we decided to apply [^18^F]AV-45 to the diagnosis of recurrent MTC with clinical PET/CT imaging.

Initially, to produce clinical-grade [^18^F]AV-45, we developed the automated radiochemical preparation, purification, and formulation of [^18^F]AV-45 on a Trasis Allinone radiosynthesizer. [^18^F]AV-45 is routinely produced in multiple patient dose with this fully automated procedure. The quality control indicates that [^18^F]AV-45 produced meets the European Pharmacopeia standard for radiopharmaceuticals. Next, we tested [^18^F]AV-45 on tissue sections from five different MTC patients and healthy human thyroid tissue samples as the negative control. Amyloid deposition was confirmed by both Congo Red and H&E staining. Localized accumulation of [^18^F]AV-45 in the tissue sections of all five MTC patients was observed. The distribution of the radioactivity in these tissue sections had similar patterns of Congo Red and H&E staining. Moreover, the radioactivity can be largely blocked by the [^18^F]AV-45 nonradioactive reference compound. In contrast, the [^18^F]AV45 had little uptake by the healthy human thyroid tissue. These results demonstrate that [^18^F]AV-45 can selectively and specifically accumulate in the MTC amyloid plaques. To ensure that [^18^F]AV-45 has no specific accumulation in the healthy neck tissues, including thyroid, lymph nodes, and muscle, we conducted the [^18^F]AV-45 PET/CT imaging study with four volunteers without thyroid disease history. Only the background level of radioactivity was observed in the thyroids, neck lymph nodes, and muscle from all four volunteers. Encouraged by these results, we compared the use of [^18^F]FDG and [^18^F]AV-45 PET/CT imaging for the detection of metastasis in a recurrent MTC patient. Several neck lymph nodes with increased [^18^F]FDG uptake were observed. Only one of them showed increased [^18^F]AV-45 uptake in the PET imaging studies. All these lymph nodes were removed by surgery. Postoperative histopathological analysis with Congo Red and H&E staining confirmed that the [^18^F]AV-45 positive lymph node had MTC metastasis with amyloid plaque deposition. In contrast, the [^18^F]AV-45 negative lymph nodes were free from MTC with no amyloid plaques detected. It is well documented in the literature that [^18^F]FDG can give false-positive diagnosis of lymph node metastasis due to local inflammation.^19,20^ In this recurrent MTC patient, [^18^F]FDG cannot differentiate the metastatic lymph nodes from the inflammation lymph nodes. [^18^F]AV-45 clearly has the specificity to detect MTC lymph node metastasis. The patient’s calcitonin level was rapidly decreased post the radical neck dissection and free from MTC at the time of this report. It is worth noting that the [^18^F]AV-45 uptake is related to the degree of amyloid deposition in the MTC. The [^18^F]FDG uptake is correlated with the expression and function of the glucose transport proteins and the intracellular hexokinase. Because of the different accumulation mechanisms, the radioactivity distributions of [^18^F]AV-45 and the [^18^F]FDG on the same MTC-positive lymph node were not completely overlapped. The key limitation of this pilot clinical study is that only one MTC patient was recruited and imaged with [^18^F]AV-45. Currently, we are preparing to expand the pilot clinical study to examine the sensitivity of [^18^F]AV-45 for MTC diagnosis with a larger patient population. We expect that [^18^F]AV-45 would be a valuable PET tracer for not only the diagnosis of MTC but monitoring the therapeutic efficacy of MTC chemo- and radiotherapies. For example, several ^111^In- and ^177^Lu-labeled cholecystokinin-2 receptor targeting minigastrin (MG) peptide analogues have shown great promise for MTC theranostic applications.^21,22^ As [^18^F]AV-45 has different molecular mechanisms for MTC uptake to these MG peptides, it would be sensible to conduct a comparison study to investigate the sensitivity and reliability between [^18^F]AV-45 and radiolabeled MG peptides to monitor peptide receptor radionuclide therapies.

## Conclusions

In this first-in-human pilot clinical study, we have demonstrated that [^18^F]AV-45 exhibits selective and specific uptake by the amyloid plaques in MTC tissue samples. [^18^F]AV-45 PET/CT imaging successfully detected MTC lymph node metastasis in a recurrent MTC patient. In contrast, [^18^F]FDG gave false-positive results for multiple lymph node metastasis. These results warrant further assessment of [^18^F]AV-45 for MTC diagnosis with PET/CT imaging.

## Supporting Information Available

AV-105 characterization:^1^ H NMR, high-resolution MS, and HPLC chromatogram. Trasis Allinone automated synthesizer with a [^18^F]AV45 cassette. Trasis Allinone automated synthesizer [^18^F]AV45 radiosynthesis module control diagram. Quality control of [^18^F]-AV-45. HPLC chromatogram of the nonradioactive reference compound (above); HPLC chromatogram of purified [^18^F]-AV-45 (below) (PDF)

## Abbreviations

[^18^F]FDOPA: 3,4-dihydroxy-6-[^18^F]fluoro-l-phenylalanine
[^18^F]FDG: 2-deoxy-2-[^18^F]fluoro-d-glucose
MRI: Magnetic resonance imaging
MTC: medullary thyroid carcinoma
MBq: megabecquerel
PET: positron emission tomography
SPECT: single-photon emission computed tomography
SUV: standardized uptake value
