# Supplementary material for: Targeting Amyloids with [18F]AV-45 for Medullary Thyroid Carcinoma Positron Emission Tomography/Computed Tomography Imaging: A Pilot Clinical Study
Source: Mol Pharm. 2022 Jan 4;19(2):584–91. doi: 10.1021/acs.molpharmaceut.1c00680 (PMC8826757; doi:10.1021/acs.molpharmaceut.1c00680)
Supplement: Supplementary file 1 — mp1c00680_si_001.pdf [file mp1c00680_si_001.pdf]

Supporting information

Targeting the Amyloid with [^{18}F]AV-45 for Medullary Thyroid Carcinoma PET/CT Imaging: A Pilot Clinical Study

Chun Li#, Pengxin Zhang†, Ruirui Nie#, Xiaoyan Gong#, Jinghui Xie#, Zilin Yu*, Chengdong Wang#, Hua Zhang, # Ran Yan*, Zhi Lu#

#Department of Nuclear Medicine, First Affiliated Hospital of Dalian Medical University, People's Republic of China, 116021;

†Department of Pathology, First Affiliated Hospital of Dalian Medical University, People's Republic of China, 116021;

*King's College London, School of Biomedical Engineering and Imaging Sciences, St. Thomas' Hospital, SE1 7EH, London, United Kingdom.

AV-105 Characterization

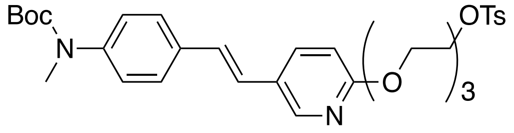

AV-105 was prepared following literature methods. [1] ^{1}H-NMR spectra was recorded on 400 MHz NMR Spectrometer instrument (Bruker Avance II, Vaian DLG400). Tetramethylsilane (δ 0.00) was used as internal standard for ^{1}H-NMR.^{1}H NMR (400 MHz, Chloroform-*d*): δ8.18 (d, *J* = 2.3 Hz, 1H), 7.82-7.77 (m, 3H), 7.44 (dt, *J* = 9.0, 2.1 Hz, 2H), 7.33 (d, *J* = 8.0 Hz, 2H), 7.23 (d, *J* = 8.5 Hz, 2H), 6.97 (d, *J* = 1.3 Hz, 2H), 6.79 (d, *J* = 8.6 Hz, 1H), 4.50-4.44 (m, 2H), 4.18-4.13 (m, 2H), 3.85-3.80 (m, 2H), 3.72-3.67 (m, 2H), 3.67-3.58 (m, 4H), 3.27 (s, 3H), 2.43 (s, 3H), 1.46 (s, 9H). HPLC conditions: XDB-C18 column (Waters XBridge Prep, 5 μm, 4.6 x 150 mm). The mobile phase: solvent A, AcCN and solvent B, H_2O; starting from 60% of A to 100% of A in 20 min and go back to 60% of A in another 10 min; with a flow rate of 1.0 mL/min. The retention time of AV-105 is 11.0 min. HRMS: calculated for $[M+Na]^+$ $C_{32}H_{40}N_2NaO_8S^+$ 635.2403, found m/z 635.1773. The data are comparable with those reported in literature.[1]

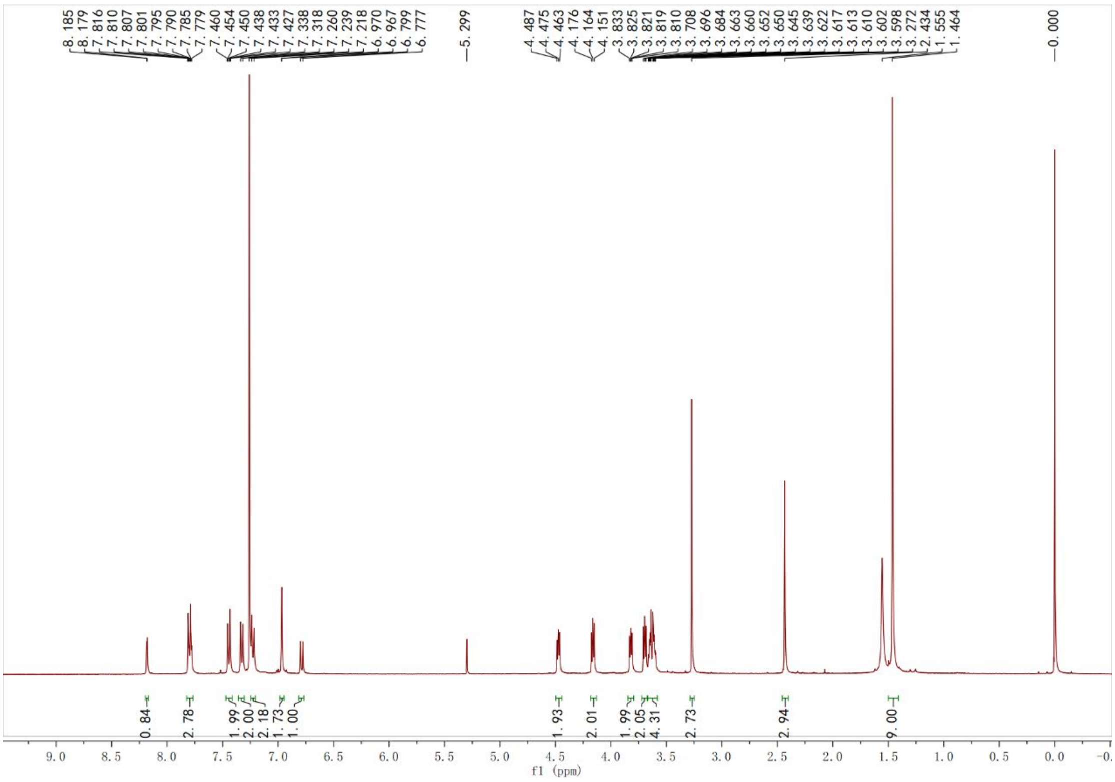

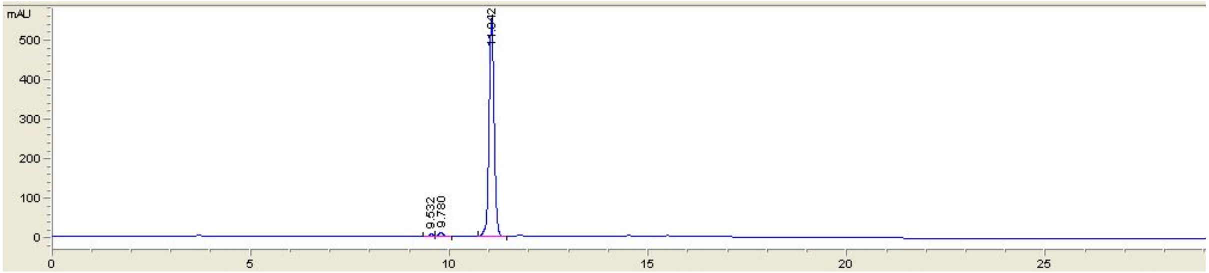

References:

[1]. Yao, T. and Z. Li, Facile synthesis of TEG-substituted 4-(N-methyl-N-Boc-amino)styrylpyridine and PET imaging agent [F]florbetapir ([F]AV-45). Synthetic Communications, 2018. 48(4): p. 422-427.

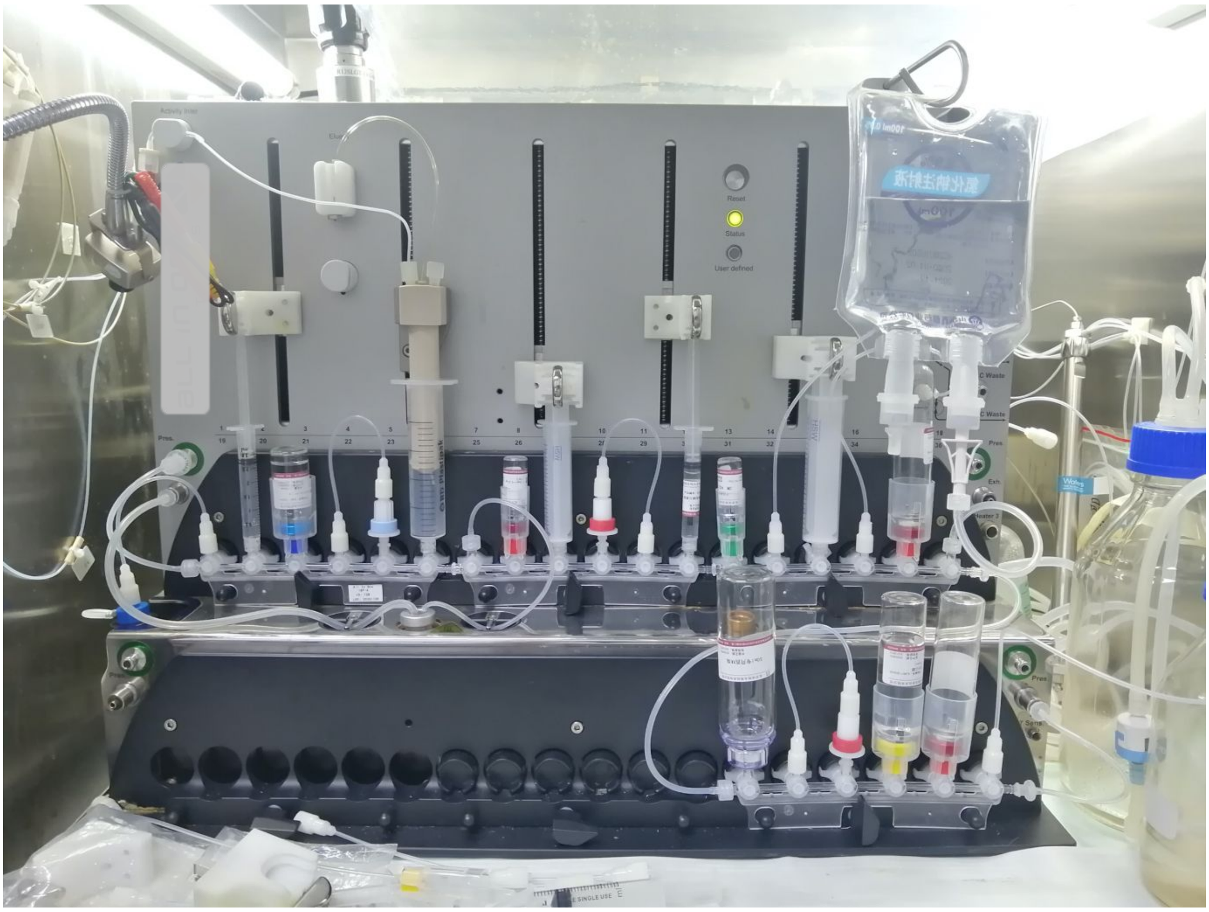

Figure S1. Trasis Allinone automated synthesizer with [^{18}F]AV45 cassette

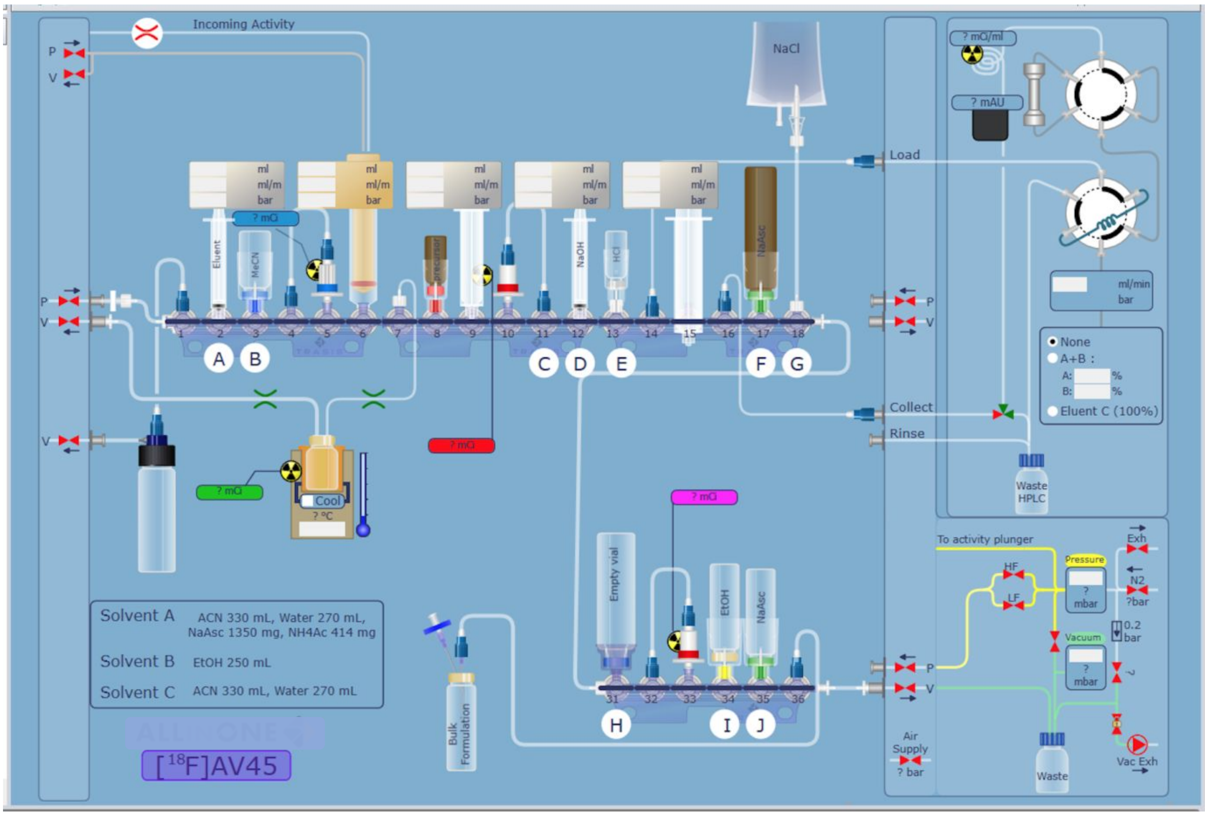

Figure S2. Trasis Allinone automated synthesizer [^{18}F]AV45 radiosynthesis module control diagram.

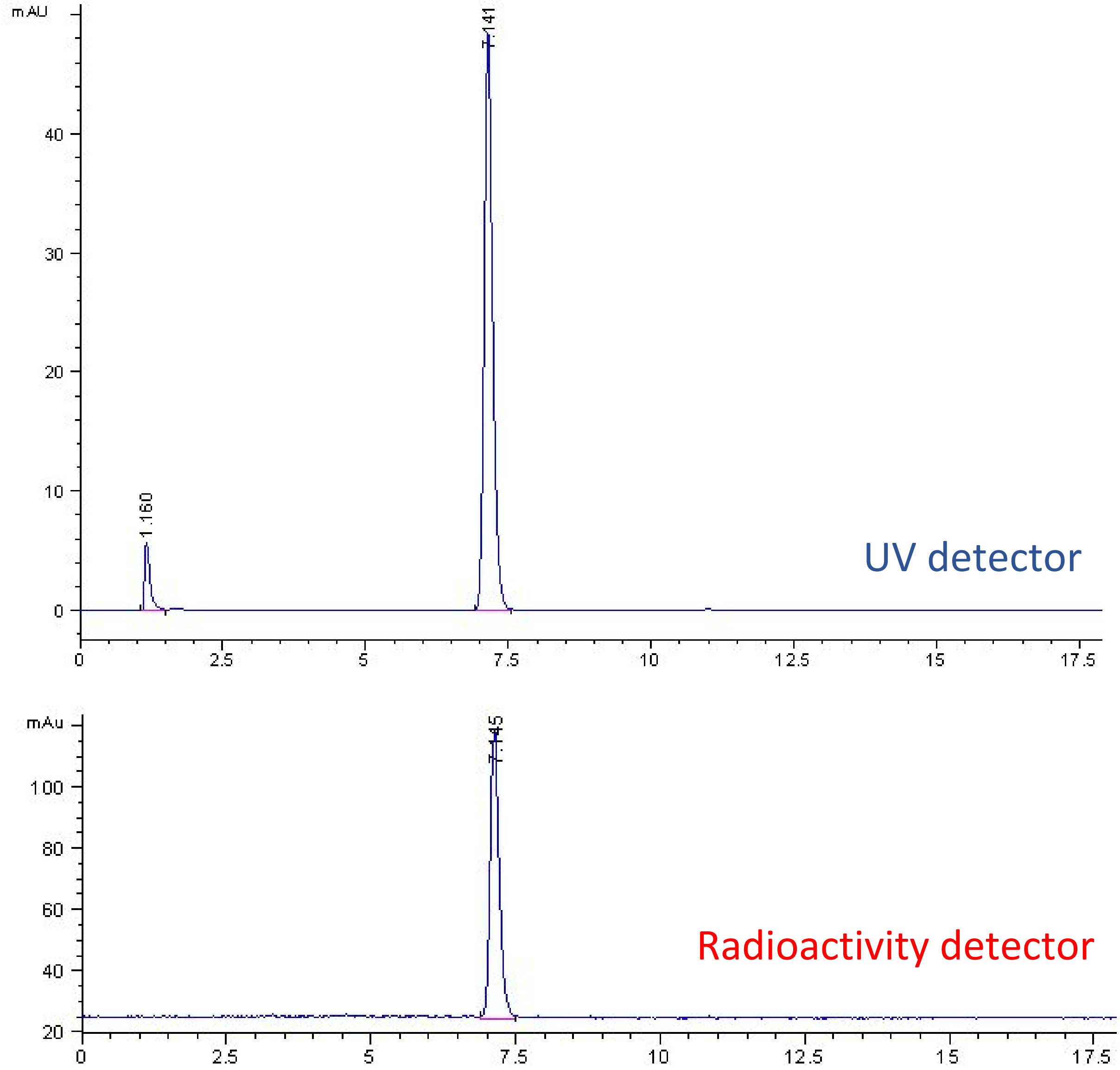

Figure S3. Quality control of [^{18}F]-AV-45. HPLC chromatogram of non-radioactive reference compound (above); HPLC chromatogram of purified [^{18}F]-AV-45 (below).
